# Development of a Novel Alginate-Based Amlodipine Nanoplex for the Formulation of an Oral Film in Antihypertensive Therapy

**DOI:** 10.3390/pharmaceutics18060653

**Published:** 2026-05-27

**Authors:** Javiera Medina, Thamara Hidalgo, Fabián Martínez, María Elena Gamboa-Arancibia, Néstor Gutiérrez-Sánchez, Sebastián Miranda-Rojas, Alexander Gamboa

**Affiliations:** 1 Departamento de Ciencias del Ambiente, Facultad de Química y Biología, Universidad de Santiago de Chile, Av. Libertador Bernardo O’Higgins 3363, Estación Central, Santiago 9170022, Chile; javiera.medina.ca@usach.cl (J.M.); thamara.hidalgo@usach.cl (T.H.); fabian.martinez@usach.cl (F.M.); maria.gamboa.a@usach.cl (M.E.G.-A.); 2 Departamento de Ciencias Químicas, Facultad de Ciencias Exactas, Universidad Andrés Bello, Av. República 275, Santiago 8370146, Chile; n.gutierrrezsnchez@uandresbello.edu (N.G.-S.); sebastian.miranda@unab.cl (S.M.-R.)

**Keywords:** alginate, nanoplexes, amlodipine, oral film, polyelectrolyte–drug interactions, amorphous solid state, computational simulations

## Abstract

**Background/Objectives:** Amlodipine is an antihypertensive agent characterized by low aqueous solubility and variable oral bioavailability. This study aimed to formulate and characterize amlodipine–alginate nanoplexes and to incorporate the optimized system into an oral film dosage form. **Methods:** Nanoplexes were prepared via ionic complexation employing alginates (ALG) with diverse physicochemical properties, including low (LV) and medium (MV)-viscosity grades, as well as alginates with varying M/G ratios. The nanoplexes were thoroughly characterized employing a comprehensive set of analytical techniques. In addition, intermolecular interactions were examined using computational simulation studies. **Results:** The nanoplexes demonstrated high encapsulation efficiencies (>80%), with MV alginate yielding particles with greater drug loading but larger mean diameters compared with that prepared using LV alginate. Computational simulation studies revealed favorable interaction energies between the drug and the polyelectrolyte, particularly within microenvironments enriched in guluronic acid–rich repeat regions. These interactions were corroborated by infrared spectroscopy, while differential scanning calorimetry and X-ray diffraction analysis confirmed the amorphous solid state of amlodipine within the nanoplexes. Dissolution studies demonstrated an inverse relationship between alginate viscosity and drug release rate, with formulations based on LV alginate exhibiting rapid drug release. The final hydroxypropylmethylcellulose film incorporating ALG-MV nanoplexes exhibited adequate mechanical integrity and achieved approximately 95% drug release within 30 min. **Conclusions:** The developed film presenting a viable approach to enhance the delivery of amlodipine. Overall, this approach constitutes a significant advancement in the delivery of poorly soluble drugs through the integration of nanostructured systems with flexible oral film platforms.

## 1. Introduction

Hypertension (HTN) is a leading contributor to premature mortality globally and constitutes a major modifiable risk factor for cardiovascular disease [1]. In Chile, the prevalence of HTN is estimated to be approximately 27% in the general population, increasing to nearly 75% among older adults [2]. HTN is currently defined by systolic blood pressure (SBP) values ≥130 mmHg and/or diastolic blood pressure (DBP) values >80 mmHg. Although diagnostic criteria and classification thresholds have evolved over time, there is broad consensus that sustained blood pressure levels ≥140/90 mmHg justify the initiation of pharmacological therapy, with a recommended treatment goal of ≤130/80 mmHg [3]. With regard to pharmacotherapy, a substantial proportion of antihypertensive agents exhibit low oral bioavailability, which can be attributed to poor aqueous solubility and/or limited intestinal permeability [4]. Amlodipine (AML) is a third-generation dihydropyridine calcium channel blocker that is widely prescribed due to its efficacy in inducing vascular smooth muscle relaxation and thereby reducing peripheral vascular resistance [5]. In its free base form, amlodipine exhibits low aqueous solubility and is therefore formulated as the besylate salt to enhance its solubility [6,7]. However, its oral bioavailability is variable, ranging from 52% to 88%, with a reported mean value of approximately 64% [8]. AML possesses unique pharmacokinetic characteristics, notably a prolonged terminal elimination half-life of approximately 40–50 h, which eliminates the necessity for modified-release formulations due to its intrinsic capacity to maintain sustained antihypertensive activity [8,9]. Consequently, an immediate-release dosage form with appropriate dissolution properties is considered adequate for once-daily administration.

Multiple formulation strategies have been developed to enhance the solubility of poorly water-soluble drugs, including micellar solubilization [10,11], cyclodextrin inclusion complexation [12,13], solid dispersions [14,15], and related approaches [16]. These methods aim to address a challenge that is estimated to affect approximately 70–90% of newly developed chemical entities [17]. In the case of AML, advanced formulation approaches such as dry emulsions [18] and solid dispersions [19] have been employed. These strategies have been shown to enhance dissolution and/or absorption, as evidenced by increases in maximum plasma concentration (C max) and area under the plasma concentration–time curve (AUC) observed in animal studies [18].

Additional strategies for advancing innovative solutions in pharmaceutical development include the application of nanotechnology-based approaches [20]. Reduction in particle size to the nanoscale markedly increases the specific surface area, thereby enhancing the dissolution rate and potentially improving bioavailability [21,22]. In the case of AML, nanostructured lipid carriers have been developed to facilitate transdermal delivery and enhance systemic bioavailability [23]. In addition, topical formulations comprising amlodipine-loaded solid lipid nanoparticles incorporated into a hydrogel matrix have been reported to enhance burn wound healing [24]. In a separate approach, amlodipine-loaded polymeric nanoparticles have been investigated as a repurposed antibacterial ocular therapy for the treatment of *Staphylococcus aureus*–induced infections [25], as well as for sustained controlled release using poly(lactic-co-glycolic acid) (PLGA) nanoparticles [26]. Additionally, AML has been adsorbed onto nanodiamonds [27] and gold nanoparticles [28], and nanocomposite systems incorporating the drug have been developed [29,30].

None of the aforementioned systems have exploited the cationic nature of AML for nanocarrier fabrication. Accordingly, the present study proposes the development of amlodipine-based nanoplexes as a novel delivery system for this drug. Compared to traditional organic solvent–dependent methodologies, including liposomes, solid lipid nanoparticles (SLNs), and PLGA-based polymeric nanoparticles, the polyelectrolyte nanocomplexation approach provides multifaceted advantages in terms of formulation efficiency and functionality.

Nanoplexes are formed via electrostatic interactions between a charged drug and an oppositely charged polyelectrolyte [31]. Their principal advantages include compliance with green chemistry principles, as complexation commonly occurs spontaneously in aqueous media without the use of harsh organic solvents [32]. Furthermore, nanoplexes typically exhibit high encapsulation efficiency and can stabilize the drug in an amorphous state, which displays substantially higher solubility than the crystalline form [33]. For cationic drugs, the fabrication of nanoplexes requires the use of a negatively charged polyelectrolyte; accordingly, materials such as dextran sulfate [34], carrageenan [35], or alginate [36] have been employed for this purpose. However, for this latter biopolymer, no studies have explored the use of different sources with distinct physicochemical properties that may influence the interaction between the drug and the polyelectrolyte during the formation of nanoplexes with varied behaviors. Moreover, in drug delivery applications, it is essential to employ excipients that are widely accepted by regulatory agencies; this supports the selection of alginate, which complies with pharmacopoeial standards and is available in various grades tailored to specific applications [37,38].

Alginate (Alg) is a naturally occurring anionic polysaccharide derived from brown algae, composed of linear copolymers containing blocks of β-D-mannuronate (M units) and α-L-guluronate (G units) residues linked through β-glycosidic bonds [39]. This polyelectrolyte has been extensively employed in carrier formation due to its ability to undergo gelation in the presence of divalent cations, such as calcium, via ionic gelation [40], as well as to form complexes through coacervation with cationic polymers [41,42]. However, reports on its direct use in the formation of nanoplexes with cationic drugs remain scarce in the literature. This study evaluated the formulation of amlodipine–alginate nanoplexes by varying key parameters, including the specific alginate type (low or medium viscosity alginate), alginate composition (low and high M/G ratios), drug-to-polymer ratios, and the incorporation of stabilizing agents such as NaCl and Pluronic F127. These systems were characterized using a comprehensive set of analytical techniques, which confirmed their nanometric dimensions, the presence of ionic drug–polyelectrolyte interactions, an amorphous solid state, and enhanced dissolution behavior. In addition, computational simulation studies demonstrated favorable interaction energies between the drug and the polyelectrolyte, particularly within microenvironments provided by G-rich repeat regions. The optimized nanoplexes were subsequently incorporated into oral film formulations as an innovative pharmaceutical dosage form, and their properties were evaluated. These thin polymeric layers are designed to rapidly disintegrate upon contact with saliva, enabling immediate release of the active pharmaceutical ingredient [43]. Moreover, oral films offer advantages over conventional dosage forms, including improved dose personalization and enhanced patient convenience [44]. In contrast to conventional tablets, which require administration with water, oral films are designed for administration without water and are particularly advantageous for pediatric and geriatric populations, as well as for patients with dysphagia [45].

## 2. Materials and Methods

### 2.1. Materials

Amlodipine (AML) was obtained from Shenzhen nexconn pharmatechs Ltd. (Shenzhen, China). Pluronic^®^ F127 (PF127) and sodium alginate (ALG), in its low-viscosity (ALG-LV) and medium-viscosity (ALG-MV) forms, were obtained from Sigma-Aldrich (St. Louis, MO, USA). Hydrochloric acid, glycerin, sodium chloride and acetic acid was purchased from Merck (Darmstadt, Germany). Sodium alginate from *Lessonia trabeculata* (IL-6G) and *Lessonia berteroana* (IL-6F) were obtained from Alginatos Chile S.A. (Santiago, Chile). Hydroxypropylmethylcellulose (HPMC)-Methocel E4M Premium was obtained from Colorcon (Harleysville, PA, USA). Purified and Milli-Q^®^ water were produced in the laboratory.

### 2.2. Drug Characterization

AML was characterized by proton nuclear magnetic resonance (^1^H-NMR) spectroscopy in deuterated dimethyl sulfoxide (DMSO-d_6_) using a Bruker Avance 400 instrument (Bremen, Germany); direct infusion electrospray ionization mass spectrometry (ESI-MS) on a TSQ Endura system (Thermo Scientific, Waltham, MA, USA); attenuated total reflectance infrared (ATR-IR) spectroscopy with a Spectrum Two spectrometer (PerkinElmer, Beaconsfield, UK); and differential scanning calorimetry (DSC) using a Setaram instrument (Cranbury, NJ, USA). For determination of drug solubility, a saturated solution of AML was prepared in water and incubated in a dual-action shaking water bath (PolyScience, Inc., Warrington, PA, USA) for 24 h at 100 cycles/min and 37 °C. Following incubation, aliquots were filtered through a 0.45 µm PVDF syringe filter (Merck Millipore, Darmstadt, Germany) and quantified by UV spectrophotometry at 239 nm using an Infinite M200 Pro plate reader (Tecan AG, Männedorf, Switzerland). Drug concentration was quantified using a calibration curve generated from serial dilutions, producing AML standards at 0.005, 0.0075, 0.010, 0.0125, 0.015, 0.0175, and 0.020 mg/mL (Correlation coefficient = 0.997).

### 2.3. Alginates Characterization

The proportion of mannuronic acid (M) to guluronic acid (G) residues (M/G ratio) in the alginates was determined by 1H NMR spectroscopy using a Bruker Avance 400 instrument (Bremen, Germany), following the method described by Belattmania et al. [46]. Approximately 10 mg of sample was dissolved in 650 µL of D_2_O at 60 °C. The solution was transferred to an NMR tube and inserted into the spectrometer. The temperature was then increased to 340 K and equilibrated for 5 min prior to acquisition. Spectra were acquired using the zg30 pulse program with 32 scans and 2 dummy scans. Chemical shifts were referenced to the residual solvent signal. The M/G ratio and sequence distribution were determined by integrating signals corresponding to monads (F_G_ and F_M_) and dyads (F_MM_, F_GG_, F_MG_, and F_GM_). For this purpose, three signals were defined within the anomeric region: A1 (δ 5.3–5.0 ppm), A2 (δ 4.9–4.7 ppm), and A3 (δ 4.7–4.5 ppm). The M/G ratio was determined according to the following expressions:

FG = A1/(A2 + A3)

FM = 1 − FG

M/G = (1 − FG)/FG

Infrared spectra were acquired over the spectral range of 400–4000 cm^−1^ using a Spectrum Two ATR-FTIR spectrometer (PerkinElmer, Beaconsfield, UK); and viscosity measurements of the alginates at 2.0% (*w*/*v*) were performed using a VISCO™ B viscometer (Atago, Tokyo, Japan). The following conditions were applied to each alginate according to the manufacturer’s recommendations: ALG-LV using an L1-type spindle at 100 rpm; ALG-MV with an L1-type spindle at 2 rpm; and IL-6F and IL-6G with an L1-type spindle at 20 rpm. At the concentration used for viscosity determination, alginate exhibits pseudoplastic behavior, as reported and validated in the literature [47]; therefore, viscosity measurements were recorded two minutes after initiating spindle rotation to ensure stable readings.

### 2.4. Preparation of AML-ALG Nanoplexes

For nanoplex preparation, aqueous solutions of amlodipine free base (AML) were formulated in 1% (*v*/*v*) acetic acid, while sodium alginate solutions were prepared in water. Two commercial alginate grades, low-viscosity (ALG-LV) and medium-viscosity (ALG-MV) were utilized at concentrations of 0.5, 1.0, and 2.0 mg/mL. AML solutions were prepared at concentrations of 0.5, 1.0, and 3.0 mg/mL. Additionally, alginates extracted from the brown macroalgae *Lessonia trabeculata* (IL-6G) and *Lessonia berteroana* (IL-6F) were evaluated.

For each formulation, one volume of the AML solution was mixed with an equivalent volume of the alginate solution, and the resulting mixtures were stirred at room temperature for 30 min to promote nanoplex formation. The influence of ionic strength on complexation was assessed by incorporating NaCl at concentrations of 0.1 M and 0.3 M into the alginate solutions. Additionally, the impact of the stabilizing agent Pluronic^®^ PF127 (0.1% *w*/*v*) was examined in alginate solutions containing NaCl at 0.1 M and 0.3 M. The resulting suspensions were purified by centrifugation at 21,000× *g* for 30 min at 14 °C using a Hermle Z32K centrifuge (Wehingen, Germany).

### 2.5. Optical Density of Nanoplexes

Once the AML–ALG nanoplexes were formed, the optical density (OD) of each formulation was measured at 600 nm using an Infinite M200 Pro plate reader (Tecan AG, Männedorf, Switzerland).

### 2.6. Encapsulation Efficiency of Amlodipine

Samples were subjected to ultrafiltration using an Amicon^®^ Ultra-4 centrifugal filter unit with a 3 kDa molecular weight cutoff (Merck, Darmstadt, Germany). The concentration of free amlodipine in the filtrate was quantified by UV spectrophotometry at 239 nm (Tecan AG, Männedorf, Switzerland) using a previously established calibration curve. Encapsulation efficiency (EE%) for the optimal nanoplex formulations was then calculated using the following equation:

$$EE\left(\%\right)=\frac{\left[Theoretical\;drug\;added\left(mg\right)-free\;drug\left(mg\right)\right]}{Theoretical\;drug\;added\left(mg\right)}\times \;100\%$$

### 2.7. Particle Size and Zeta Potential

Measurements were conducted using a Zetasizer Nano ZS-90 instrument (Malvern Panalytical Ltd., Malvern, UK). For analysis, 0.1 mL of each nanoplex suspension was diluted with 1 mL of Milli-Q water and transferred into a folded polystyrene capillary cell using a syringe. All measurements were performed in triplicate at room temperature using a 633 nm laser source.

### 2.8. Physical Characterization of Nanoplexes

Nanoplex dispersions (30 mL) were centrifuged at 21,000× *g* for 30 min at 14 °C using a Hermle Z32K centrifuge (Wehingen, Germany), followed by three cycles of resuspension in water to separate the free drug. The samples were then frozen at −80 °C and subsequently lyophilized using an IlShin FD5508 freeze dryer (IlShinBioBase Co., Ltd., Dongducheon, Republic of Korea) for 48 h. The freeze-dried samples were placed directly on the ATR crystal and analyzed using a Spectrum Two infrared spectrometer (PerkinElmer, Beaconsfield, UK). For each sample, 60 scans were collected over the spectral range of 400–4000 cm^−1^. Thermoanalytical curves were acquired using a DSC131 differential scanning calorimeter (Setaram Inc., Cranbury, NJ, USA). Approximately 8 mg of each lyophilized sample was placed in sealed aluminum crucibles and heated from 30 to 190 °C at a rate of 10 °C/min under a nitrogen purge. Crystallinity was evaluated using a Bruker D8 Advance instrument (Bruker, Bremen, Germany) with Cu Kα radiation (λ = 1.5406 Å, 40 kV, 40 mA). Each sample was uniformly distributed on a Lucite sample holder, and diffraction patterns were recorded over an angular range of 2–50° (2θ).

### 2.9. Dissolution Profile of Nanoplexes

Ten milligrams of AML and lyophilized nanoplexes, prepared as described in Section 2.8, were dispersed in 100 mL of water and maintained in a dual-action shaking water bath (PolyScience, Inc., Warrington, PA, USA) at 100 cycles/min and 37 °C. Aliquots were withdrawn at 10, 20, 30, 40, 60, 90 and 120 min, and an equivalent volume of fresh water was added to the dissolution vessel. Each aliquot was subsequently filtered through a 0.45 µm PVDF syringe filter (Merck Millipore, Darmstadt, Germany). AML concentration in the filtrates was quantified by UV spectrophotometry at 239 nm, as previously described.

### 2.10. Molecular Modeling of the AML-ALG Complex

To consider all possible combinations of mannuronic (M) and guluronic (G) acid residues within the three-subunit models, six alginate (ALG) block configurations were constructed and, for simplicity, designated as GGG, GMM, GMG, MGM, GGM, and MMM. The initial coordinates for AML and ALG models were designed using the Avogadro v1.2.0 package [48]. The complexes for each ALG model were generated by locating the positively charged ammonium group from AML near one of the three carboxylate group from ALG, generating three models per ALG, one for each carboxylate group, with a total of eighteen starting structures. To properly discriminate the lowest energy complex, each model used as starting coordinates was subjected to extensive conformational sampling by using the crest subroutine [49], assisted by the quantum semiempirical approach provided by the x-TB software v6.5.1 [50]. The crest program performs an accelerated molecular dynamics simulation based on metadynamics, by which an exhaustive search of minimum energy conformations is carried out, optimized and sorted by energy. Three ensembles of conformations per ALG models were generated. These ensembles were combined for each model, and the three conformations with the lowest energy were selected, and preoptimized by means of DFT using BP86 as the exchange-correlation functional [51], coupled with a def2-SVP basis set for all atoms. The preoptimizations were carried out using the Turbomole v7.3 program [52]. The lowest energy conformation was selected and optimized with the ADF package v2022.102 [53], at the DFT level of theory using B3LYP [54] with a TZ2P basis set. Solvent effects were incorporated at this stage through the implicit solvation model COSMO [55]. These models were used for further analyses.

The interaction energies (∆E_int_) were calculated using the energies from the optimized geometries of the individual models for ALG, AML, and the optimized complexes, energies that were used according to the following equation:

$${∆E}_{int}=E_{complex}-E_{ALG}-E_{AML}$$

The characterization of the energetic contributions responsible of the differential affinity of ALM to different microenvironments provided by the ALG polymer were performed by using the energy decomposition analysis (EDA). This method allows to decompose the interaction energy into its main physical contributions, providing a basis for the rationalization of the driving forces behind the interaction strength that leads to complex formation. The EDA scheme implemented in ADF contemplates the decomposition of the interaction energy according to the following equation:

$${∆E}_{int}={{∆E}_{oi}\left(\xi \right)+∆V}_{elstat}+{∆E}_{Pauli}+{∆E}_{disp}$$

The ∆E_oi_ term includes the contributions from the charge transfer process consequence of the orbital overlap between the fragments, and the electronic density reorganization within each fragment as part of the polarization caused by the formation of the complex. The ∆E_elstat_ contemplates the electrostatic attraction between the electronic density clouds of the fragments, while the ∆E_disp_ represents the energetic contribution coming from the dispersion forces taking part on the interaction strength. The ∆E_Pauli_ stands for the steric electronic effect that arise from the Pauli exclusion principle, and is the mains source of destabilizing effects in the formation of a complex.

The noncovalent index (NCI) [56] identifies noncovalent interactions such as van der Waals, hydrogen bonds and steric repulsion. We used NCI analysis to obtain a real space representation of noncovalent interactions between AML and the ALG microenvironments represented by the ALG models. The NCI is represented by color coding: blue for strong stabilizing interactions (such hydrogen bond), green for weak interactions (such as van der Waals), and red for repulsive interactions (such as steric repulsive).

### 2.11. Preparation of Oral Films Containing AML Nanoplex

Oral films containing AML–ALG nanoplexes were prepared by the solvent-casting method. The formulation comprised HPMC, 1.0% *w*/*v*, glycerin (0.2% *v*/*v*), and AML–ALG nanoplexes (60% *v*/*v*), with purified water added to adjust the final volume to 100 mL. HPMC was initially dispersed in water and allowed to hydrate under magnetic stirring at 300 rpm. Subsequently, glycerin and the AML–ALG nanoplexes were added to obtain a homogeneous casting solution. Aliquots (25 g) of the solution were cast into Petri dishes (9 cm) and dried in a convection oven at 40 °C for 24 h. The resulting films were stored in sealed aluminum pouches at room temperature until further characterization.

### 2.12. Characterization of Oral Films

#### 2.12.1. Thickness Measurement

Film thickness was measured at the four corners and the center of each specimen using a digital micrometer.

#### 2.12.2. Weight Uniformity

Ten film specimens (2 × 2 cm) were individually weighed using an analytical balance, and the mean weight, standard deviation, and relative standard deviation were calculated.

#### 2.12.3. Disintegration Time

A 2 × 2 cm film specimen was immersed in 10 mL of purified water at room temperature (25 ± 2 °C) under continuous stirring, and the time to film rupture was recorded using a digital stopwatch.

#### 2.12.4. pH Determination

The pH of the aqueous medium obtained following the disintegration test was determined using a calibrated pH meter.

#### 2.12.5. Mechanical Properties

Mechanical properties of the films, namely tensile strength (TS) and elongation at break (EB), were evaluated using a TA1 texture analyzer (Lloyd Instruments Ltd., Bognor Regis, UK) equipped with a 50 N load cell, following the procedure described by Chou et al. [11]. The films were cut into rectangular strips (effective testing area = 40 × 10 mm) using a precision blade, and the exact width of each specimen was verified with a digital caliper to ensure accuracy. Each film sample was clamped securely in the instrument, and the upper clamp applied a tensile force at a constant rate of 0.5 mm/s until rupture. The thickness of each film was measured using a digital micrometer (Section 2.12.1). All measurements were conducted using Nexygen Plus 3 software, and the data were analyzed using stress–strain curves. Tensile strength (TS) and elongation at break (EB) were calculated according to the following equations:

$$TS=\frac{Peak\;stress}{Cross\text{-}sectional\;area\;of\;the\;sample}$$

$$EB=\frac{Increase\;in\;length\;at\;break}{Original\;film\;length}\times 100$$

### 2.13. Dissolution Testing of Amlodipine Polymeric Films

The dissolution test was performed using a paddle-over-disk system (USP Apparatus 5) on a Pharmatest dissolution apparatus (Hainburg, Germany). The conditions specified in the USP monograph for Amlodipine tablets [57] were employed, consisting of 500 mL of 0.01 N HCl maintained at 37 °C and agitated at 75 rpm. Samples were withdrawn at 5, 10, 15, 20, and 30 min, filtered through 0.45 µm PVDF membranes, and analyzed by UV spectrophotometry at 239 nm. Quantification was performed using a calibration curve prepared with AML standards at concentrations of 0.002, 0.004, 0.006, 0.008, and 0.010 mg/mL in 0.01 N HCl, yielding a correlation coefficient of 0.991.

### 2.14. Statistical Analysis

All quantitative experiments were conducted in triplicate, and the results are reported as mean values ± standard deviation (SD). One-way analysis of variance was applied, as appropriate, to assess statistical significance (*p* < 0.05).

A similarity test will be conducted to compare profiles and evaluate differences among dissolution curves, using the similarity factor (f_2_). This factor is defined as the logarithmic reciprocal square root transformation of the sum of squared errors and is calculated according to the following equation:

$$f_{2}=50\log\left({\left[1+\frac{1}{n}\sum_{t=1}^{n}{\left(R_{t}-T_{t}\right)}^{2}\right]}^{-0.5}\times 100\right)$$

Here, n represents the total number of sampling points, *R_t_* denotes the percentage of drug dissolved at time t for one profile, and *T_t_* corresponds to the percentage dissolved at the same time point for the other profile. In this context, an f_2_ value greater than 50 indicates that the mean difference between the two curves does not exceed 10% at the sampled time points, suggesting that the dissolution profiles are similar. Six measurements were considered in total, with only one taken after the drug reached 85% dissolution, in order to minimize statistical bias.

## 3. Results

### 3.1. Drug Characterization

Amlodipine in its free-base form is not described in compendial monographs such as the United States Pharmacopeia; therefore, comprehensive physicochemical characterization was performed to confirm its identity. Table 1 summarizes the analytical tests performed. The chemical structure of the drug was confirmed by proton nuclear magnetic resonance spectroscopy (Figure S1), as the number of protons, their integration values, multiplicity patterns, and chemical shifts were consistent with the assigned molecular structure. In the ATR-IR spectrum (Figure S2), characteristic absorptions were observed for N–H stretching (3389 cm^−1^), carbonyl stretching vibration (1682 cm^−1^), N–H bending (1646 cm^−1^), C=C stretching (1601–1473 cm^−1^), C–N stretching (1308 cm^−1^), ester (C–O) asymmetric-symmetric stretches (1276–1202 cm^−1^) and ether (C–O–C) symmetric stretch (1035 cm^−1^). Mass spectrometric analysis (Figure S3a) showed a parent ion peak with a difference in only 0.03 Da between the experimental and theoretical masses. The compound was identified as a crystalline solid exhibiting an endothermic event corresponding to its melting (Figure S3b). Additionally, the drug demonstrated low aqueous solubility (0.11 ± 0.01 mg/mL).

### 3.2. Alginates Characterization

Table 2 summarizes the physicochemical properties of the different alginates used in this study. The M/G ratio was highest for alginates derived from *Macrocystis pyrifera*, indicating a predominance of mannuronic (M) acid residues; alginates with high M content typically form softer and more elastic gels [58]. IL-6F also exhibited a high M/G ratio and can be classified within this group. In contrast, IL-6G was enriched in guluronic (G) acid, with an M/G ratio of 0.62. Alginates with high G content are known to form more rigid, brittle, and mechanically stronger gels [59]. All four samples exhibited highly similar infrared absorption profiles, with bands observed at 1596–1599 cm^−1^ corresponding to the asymmetric stretching vibrations of carboxylate groups and at 1404–1407 cm^−1^ corresponding to the symmetric stretching vibrations of carboxylate groups [46]. These features are characteristic of alginate salts. The absorption bands at 947, 902, 809 and 779 cm^−1^ observed in IL-6G are indicative of homopolymeric polyguluronic acid (GG) blocks [60]. In contrast, alginates with higher M/G ratios exhibited bands around 944 and 881–884 cm^−1^, which are characteristic of mannuronic acid (MM) blocks [46]. The consistency of these bands across all samples confirms that, despite differences in source and viscosity, the alginates share the same fundamental chemical backbone.

Regarding viscosity, three distinct alginate categories were identified. The low-viscosity alginate (ALG-LV) exhibited minimal viscosity, whereas the medium-viscosity alginate (ALG-MV) was more than two orders of magnitude more viscous than the LV variant. Alginates derived from *Lessonia* species (IL-6F and IL-6G) showed intermediate viscosities (140.9 and 183.7 mPa·s, respectively), positioning them between the LV and MV *Macrocystis* alginates.

### 3.3. Optical Density of Nanoplexes

Optical density (OD) at 600 nm is commonly used as an indirect indicator of turbidity; in this context, increased turbidity reflects the formation of colloidal particles. The literature describes systems in which turbidity is correlated with particle size in specific application [61]. In the present study, however, this technique was primarily used as a screening tool, offering a rapid and high-throughput method for monitoring nanoplex formation in a 96-well microplate format. This approach was complemented by particle size distribution analysis of selected formulations using the Zetasizer Nano ZS-90, which, although more detailed, is more time-consuming and requires more extensive sample preparation. An initial concentration range of 0.5–3 mg/mL was selected for the screening experiments based on preliminary studies. At concentrations below 0.5 mg/mL, the critical aggregation concentration is not attained, leading to unstable and erratic optical density measurements at 600 nm. Conversely, concentrations above 3 mg/mL result in rapid precipitation, followed by sedimentation of the aggregates.

**Figure 1:**
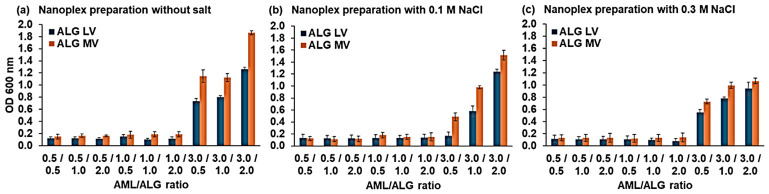
Optical density (OD 600 nm) measurements for amlodipine (AML) nanoplex formation using low-viscosity (ALG-LV) and medium-viscosity (ALG-MV) alginate in water (**a**), in 0.1 M NaCl (**b**), and in 0.3 M NaCl (**c**).

As shown in Figure 1, at the lowest AML concentration (0.5 mg/mL), all alginate combinations exhibited OD values below 0.1, suggesting minimal nanoplex formation. When the AML concentration was increased to 1.0 mg/mL, OD values approached 0.2, and the dispersions displayed a translucent appearance with adequate physical stability. Upon further increasing the AML concentration to 3.0 mg/mL, a pronounced rise in turbidity was observed, with OD values ranging from 0.7 to 1.8. At this stage, the dispersions exhibited an opalescent appearance and became unstable, leading to particle sedimentation.

Regarding the influence of alginate type (LV versus MV), medium-viscosity alginate consistently produced higher OD values than low-viscosity. For instance, at the 3.0/2.0 AML/ALG ratio, the OD of MV alginate approached 1.9, whereas that of LV alginate was approximately 1.25. These results suggest that medium-viscosity alginate, characterized by longer polymer chains, forms larger complexes, leading to increased light scattering relative to the low-viscosity variant.

Nanoplex formation in the presence of salt remained strictly dependent on surpassing a specific drug concentration threshold. The addition of 0.1 M NaCl (Figure 1b) did not alter the overall formation pattern; however, it reduced the magnitude of the response. Complexes generated under saline conditions exhibited lower turbidity, indicating the formation of stable particles. This effect is consistent with ionic shielding, in which NaCl attenuates the electrostatic interactions between the cationic drug (AML) and the anionic alginate. When 0.3 M NaCl was evaluated, a further reduction in optical density was observed, with a pronounced suppression of nanoplex formation at AML concentrations of 0.5–1.0 mg/mL. This outcome indicates that higher ionic strength exerts a stronger shielding effect, substantially diminishing the electrostatic interactions between the drug and the polymer. Under these conditions, significant nanoplex formation occurred only at an AML concentration of 3.0 mg/mL for both alginate types (LV and MV).

**Figure 2:**
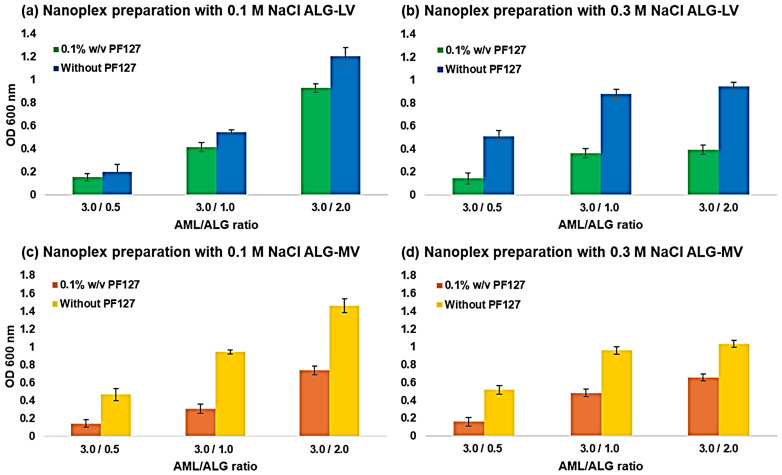
Optical density (OD 600 nm) measurements for nanoplex formation using ALG-LV with 0.1 M NaCl (**a**) and 0.3 M NaCl (**b**), and ALG-MV with 0.1 M NaCl (**c**) and 0.3 M NaCl (**d**), in the absence and presence of Pluronic F127 (PF127, 0.1% *w*/*v*).

To enhance the stability of nanoplexes prepared with AML at 3 mg/mL, the influence of Pluronic^®^ F127 (PF127) was assessed in conjunction with the effects of salt at concentrations of 0.1 M and 0.3 M. As shown in Figure 2a,b for ALG-LV, the addition of PF127 resulted in a reduction in optical density across all drug-to-polymer ratios in the presence of 0.1 M and 0.3 M NaCl. This promoted the formation of nanoparticles with enhanced stability. For ALG-MV (Figure 2c,d), PF127 produced a similar effect, decreasing the optical density of the nanoplex suspensions irrespective of whether the salt concentration was moderate or high.

### 3.4. Encapsulation Efficiency, Particle Size and Zeta Potential of Nanoplexes

Based on the optical density measurements, the most stable formulations were selected for further characterization. Table 3 presents data for nanoplexes formed between AML and ALG at different drug-to-polymer mass ratios, including the effects of NaCl and Pluronic F127 0.1% *w/v* (PF127), for both LV and MV alginates. Nanoplexes prepared with alginates extracted from *Lessonia* species at varying M/G ratios, using an AML/ALG mass ratio of 1:2, are also presented.

The overall trend indicated that all formulations achieved high encapsulation efficiencies, ranging from 80.1% to 89.5%. Increasing the proportion of alginate consistently enhanced encapsulation efficiency. For ALG-LV, increasing the drug-to-polymer ratio from 1:1 to 1:2 resulted in an increase in EE from 80.1% to 84.5% (*p* < 0.05), likely due to the greater availability of binding sites and matrix material provided by the higher polymer concentration. AML/ALG-MV 1:2 produced slightly higher encapsulation efficiencies than AML/ALG-LV 1:2. Increasing the NaCl concentration from 0.1 M to 0.3 M resulted in a modest reduction in encapsulation efficiency, likely due to competitive interactions between salt ions and the drug for binding sites on the polymer. The highest encapsulation efficiency (89.5%) was obtained for the AML/ALG-MV formulation at a 3:2 ratio in the presence of 0.1 M NaCl and PF127.

The nanoplexes exhibited particle sizes ranging from approximately 302.2 to 539.8 nm. Consistent with expectations, formulations prepared with ALG-MV yielded larger particles than those prepared with ALG-LV at comparable drug-to-polymer ratios. This behavior is attributed to the longer polymer chains and greater hydrodynamic volume of ALG-MV. In addition, increasing the alginate content led to an increase in particle size. Notably, elevating the salt concentration from 0.1 M to 0.3 M resulted in a reduction in particle size, likely due to compression of the electrical double layer and enhanced polymer chain coiling, which promotes the formation of more compact particles. With respect to polydispersity index (PDI), values ranged from 0.195 to 0.438, indicating a moderately uniform particle size distribution. Formulations containing NaCl and PF127 exhibited lower PDI values, reflecting improved homogeneity, compared with the binary drug–polymer systems.

The zeta potentials of the nanoplexes were negative, ranging from −24.4 to −38.3 mV. Increasing the alginate proportion generally resulted in more negative zeta potential values, attributable to the greater abundance of negatively charged carboxylate groups from alginate at the particle surface. Formulations prepared with ALG-MV exhibited slightly more negative surface charges than those prepared with ALG-LV, which may contribute to the enhanced colloidal stability. Zeta potential values more negative than −30 mV are commonly associated with good colloidal stability due to electrostatic repulsion that limits particle aggregation.

With respect to nanoplexes prepared using alginates derived from *Lessonia* species, IL-6G exhibited a higher encapsulation efficiency than IL-6F (89.2% vs. 85.3%) and yielded smaller particle sizes (322.0 nm vs. 394.8 nm). No significant differences in zeta potential were observed between the two formulations, with both displaying negative surface charges below −30 mV.

### 3.5. Physical Characterization of Nanoplexes

**Figure 3:**
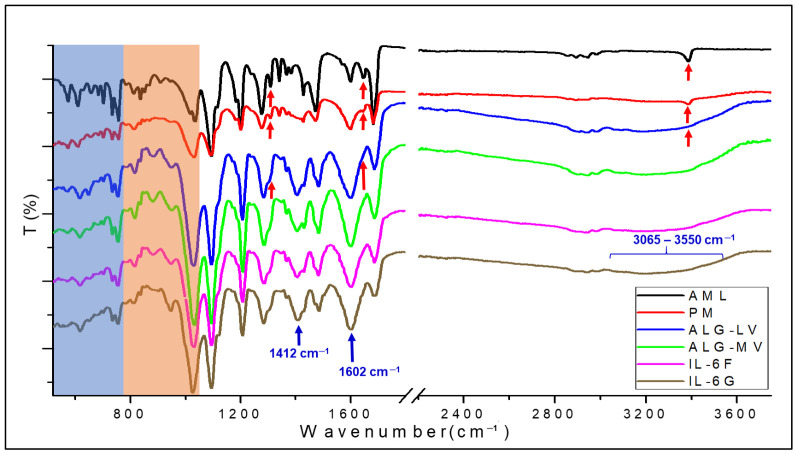
Infrared spectroscopy of AML (black), physical mixture (red), ALG-LV (blue), ALG-MV (green), IL-6F (pink), and IL-6G (brown) nanoplexes.

The IR spectrum presented in Figure 3 (Black line) displays the characteristic absorption bands of AML as previously described. The physical mixture (PM), shown in red, exhibited absorption features attributable to both alginate and AML, without evidence of significant spectral shifts or modifications. Nanoplex formation was characterized by the disappearance of absorption bands associated with the amino group of AML (3398, 1647 and 1309 cm^−1^), which are indicated by red arrows in the spectrum of the AML and PM. As a consequence of electrostatic interactions, shifts in the carboxylate absorption bands to 1412 and 1602 cm^−1^ were observed in all spectra of the complexes (blue, red, green and brown). The presence of hydrogen-bonding interactions within the formed complexes was indicated by a broad absorption band observed in the 3065–3550 cm^−1^ region. In the fingerprint region (highlighted in blue), characteristic amlodipine bands corresponding to C–Cl and aromatic C–H vibrations exhibited shifts from 573 to 570 cm^−1^ and from 758 to 754 cm^−1^, respectively. The differences observed in the orange-highlighted region are attributed to variations in the M/G ratio among the alginates used for complex formation. The main spectral change corresponded to a shift in the symmetric C–O–C stretching vibration from 1030 to 1026 cm^−1^.

**Figure 4:**
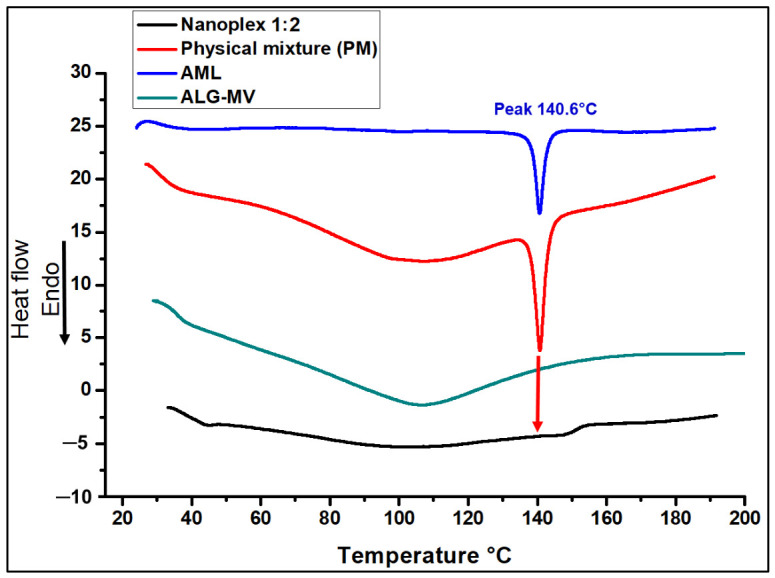
DSC of AML (blue), physical mixture (red), ALG-MV (green) and AML/ALG-MV 1:2 ratio nanoplex (black).

As shown in Figure 4, the thermogram of amlodipine (blue curve) exhibits a pronounced endothermic peak at approximately 140,6 °C, consistent with the melting point reported in the literature [62]. In contrast, the thermogram of sodium alginate (ALG-MV) (green curve) does not display a distinct melting peak, in agreement with the amorphous nature of the biopolymer; however, a broad endothermic event around 100 °C is observed, which can be attributed to the loss of bound water [63,64]. During the initial heating cycle, the glass transition temperature (Tg) of alginate may be masked by dehydration effects, as moisture acts as a plasticizer, softening the thermal baseline and thereby hindering the clear identification of a distinct Tg transition. The thermogram of the AML–ALG physical mixture (red trace) exhibited a broad endothermic event associated with the desolvation of bound water within the polymer, followed by the characteristic melting peak of amlodipine. This thermal behavior suggests the absence of significant drug–polymer interactions, as the crystalline structure of amlodipine remains unaltered. Finally, in the thermogram of the AML/ALG MV 1:2 ratio nanoplex (black trace), the characteristic melting endotherm of amlodipine was no longer observed, and the profile was dominated by low-enthalpy events attributed to residual moisture. The absence of the AML fusion peak indicates disruption of its crystalline structure, suggesting that the drug is present in an amorphous state as a result of interaction with the polyelectrolyte. Similar thermal behavior was observed for the other optimized ratios of lyophilized nanoplexes.

**Figure 5:**
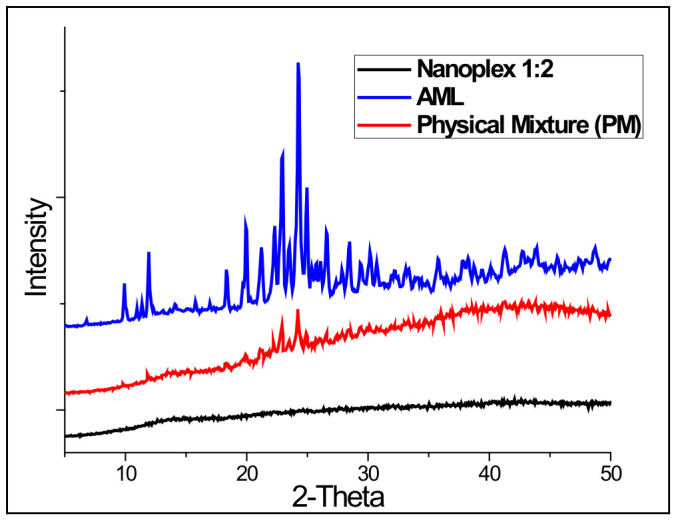
X-ray diffraction analysis of AML (blue), physical mixture (red) and AML/ALG-MV 1:2 ratio nanoplex (black).

The X-ray diffraction (XRD) patterns depicted in Figure 5 provide insight into the crystalline nature of the drug in its unprocessed form as well as following nanoplex formation. The XRD of raw amlodipine (blue trace) revealed multiple sharp and high-intensity diffraction peaks across the 2θ range, particularly within 10° to 30°, confirming the presence of a highly ordered and well-defined crystalline structure. The physical mixture (PM), prepared by manually blending (AML with MV alginate) in the absence of chemical processing, exhibited a composite X-ray diffraction pattern (red trace). This pattern is characterized by a broad diffuse halo associated with the amorphous nature of the alginate matrix, together with residual crystalline peaks of AML, including a prominent peak near 24° (2θ). Despite the reduction in peak intensity attributed to dilution effects, the persistence of these distinct sharp peaks indicates that the crystalline structure of the drug is largely retained following simple physical mixing. In contrast, the complete absence of characteristic crystalline diffraction peaks of amlodipine in the nanoplex sample provided compelling evidence of its complete amorphization (black trace). This finding indicates that the drug is no longer arranged within a well-defined crystalline lattice, but is instead molecularly dispersed in a disordered form within the ALG-MV nanoplexes.

### 3.6. Dissolution Profile of Nanoplexes

**Figure 6:**
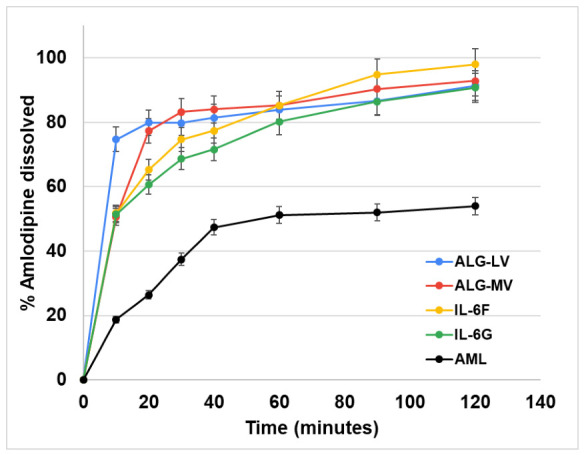
Dissolution profiles of AML (black), ALG-LV (blue), ALG-MV (red), IL-6F (orange), and IL-6G (green) nanoplexes.

The dissolution profiles of amlodipine nanoplexes and the free drug are presented in Figure 6. AML exhibited limited dissolution, with approximately 50% of the drug released after 60 min, reflecting its low aqueous solubility. In contrast, nanoplexes formulated with ALG-LV (blue line) formed a polymeric matrix that underwent more rapid dissolution, resulting in accelerated drug release and a pronounced initial burst effect. On the other hand, nanoplexes formulated with ALG-MV (red line) displayed a slower drug-release profile compared with those prepared using ALG-LV, with approximately 80% of the drug released after 30 min.

With respect to alginates derived from *Lessonia* species, nanoplexes formulated with IL-6G (*L. trabeculata*) exhibited a slower drug-release profile compared with those prepared using IL-6F (*L. berteroana*). Although both alginates exhibit intermediate viscosities, the higher viscosity and increased guluronic acid (G) content of IL-6G likely contribute to a more sustained drug release compared with IL-6F.

The similarity factor (f_2_)-based comparison of dissolution profiles (Table 4) revealed f_2_ values of 18–25 for AML relative to all nanoplexes. Values below 50 indicate a lack of similarity, confirming that the dissolution behavior of the pure drug differs markedly from that of the drug–alginate polyelectrolyte systems. The f_2_ value for ALG-LV versus IL-6G was 43, reflecting the structural extremes of these systems. ALG-LV, characterized by low viscosity and a highly open molecular structure, facilitates near-instantaneous drug release. In contrast, IL-6G, with its high guluronic acid (G) content, forms a rigid polymeric network that results in a markedly more sustained and slower drug release. A comparable trend was observed for *L. berteroana* alginate (IL-6F), with an f_2_ value of 46 when compared to ALG-LV. Meanwhile, the ALG-LV and ALG-MV pair, both obtained from *Macrocystis pyrifera*, exhibited an f_2_ value at the borderline similarity threshold, indicating that variations in viscosity affect early-stage matrix resistance.

### 3.7. Molecular Models of the Complex Between AML and ALG

**Figure 7:**
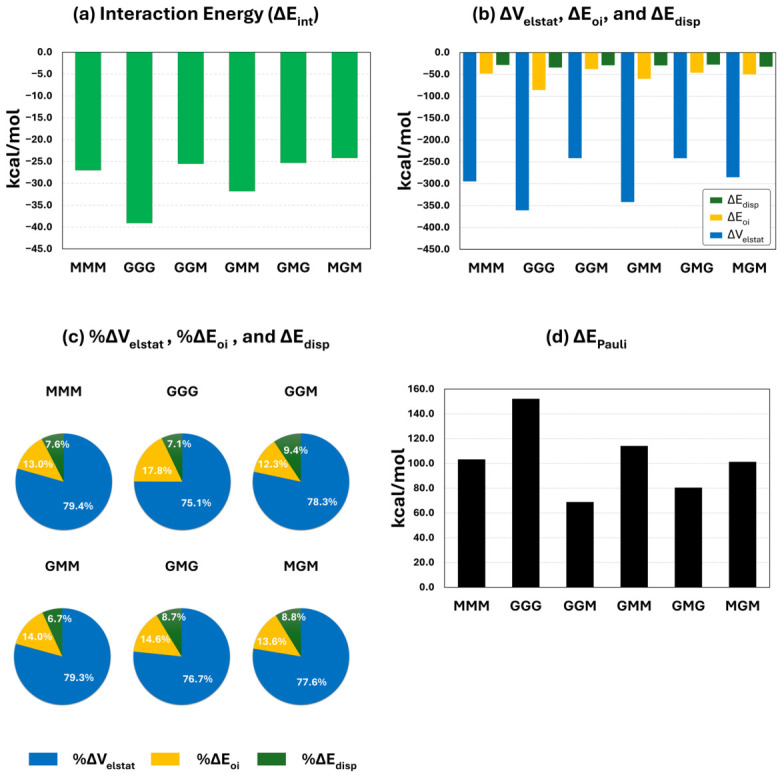
Interaction energies analysis between AML and the ALG models. (**a**) Magnitudes of the interaction energies. (**b**) Magnitudes of the stabilizing contributions to the interaction energies obtained from the EDA. (**c**) Percentage representation of the stabilizing contributions from EDA. (**d**) Magnitudes for the Pauli repulsion as a representation of the steric electronic effects.

The formation of nanoplexes using alginates with different M/G ratios resulted in measurable differences in encapsulation efficiency, particle size, and dissolution behavior. These differences can be attributed to the development of distinct microenvironments at the interaction interface between AML and the ALG matrix. To reveal the molecular features that determine the differential affinity between the different compositions of M/G subunits and AML, we designed six alginate models of three subunits each by consider all possible permutations among the subunits. The resulting interaction energies (∆E_int_) are schematically represented in Figure 7a.

**Figure 8:**
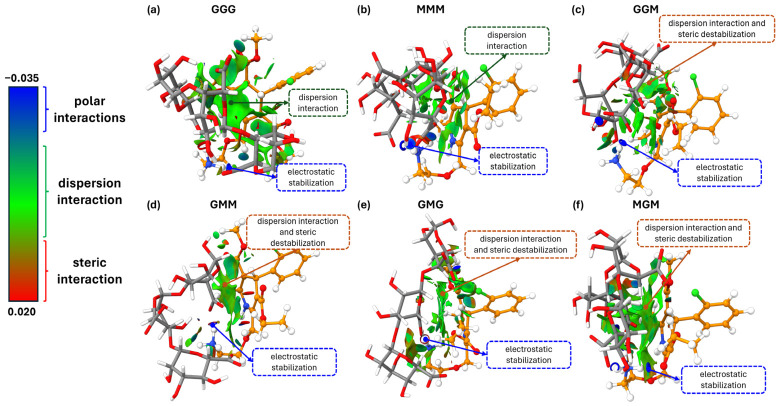
Schematic representation of the non-covalent interactions index (NCI) taking place at the interphase between AML and the ALG model. Surfaces represent the interactions taking place in real space.

The lowest ∆E_int_ was calculated for MGM with −24.2 kcal/mol, whereas the highest corresponds to GGG with −39.1 kcal/mol. The models for MMM, GGM, GMG, and MGM showed similar ∆E_int_ values with magnitudes of −27.0, −25.5, −25.3, −24.2 kcal/mol; respectively. Meanwhile, GMM showed a ∆E_int_ of −31.8 kcal/mol, with a magnitude in between GGG and the rest of the models. From an energetic point of view, ∆E_int_ is decomposed into its main physical contributions by the EDA scheme, results shown in Figure 7b,c. According to these, the interaction strength is dominated by the electrostatic contribution (∆V_elstat_), where GGG showed the strongest ∆V_elstat_ with −360.6 kcal/mol. The electrostatic interaction between the protonated amine positively charged from AML, with the carboxylate groups negatively charged from the ALG polymer, is the driving force behind the formation of the ALG nanoplexes. The percentage contribution of ∆V_elstat_ to the interaction energy was over 75.1% reaching 79.3% for GMM. Second to the electrostatic contribution regarding the contribution to ∆E_int_, is the orbital part of the interaction (∆E_oi_). This involves the polarization of the interacting parts as a stabilizing effect caused by the complex formation, and the charge transfer process coming from the potential orbital overlap in the complex conformation. The magnitudes of ∆E_oi_ varied between −85.6 kcal/mol for GGG to −37.8 kcal/mol for GGM. The percentage of contribution of ∆E_oi_ ranges between 12.3% and 17.8% for GGG, being the second most important contribution to the interaction. The charge transfer analysis provided by the NPA scheme pointed out that this is not the main contribution to the ∆E_oi_, as no charge transfer takes place, leaving to the polarization effects the lead role into the orbital contribution, which seems to be favored with GGG. The Pauli repulsion (∆E_Pauli_) is reported in Figure 7d, and it represents the main destabilizing contribution to ∆E_int_, capable of exposing the magnitude of the steric electronic effects taking place in the AML-ALG complex. The dispersion interaction corresponds to the lowest contribution, in line with its weak nature. However, it is an important indicator of the complementarity between the conformations of AML and ALG and how these fit together. These short-range interactions are depicted in Figure 8, where the non-covalent interaction index (NCI) is represented through surfaces indicating the regions where dispersion forces are present.

In terms of the specific interactions, these are graphically depicted with their corresponding distances in Figure S6 from the supporting information. MMM, GGG, GMM, and MGM models interact with AML through two electrostatic interactions between the ammonium group of AML and the carboxylate groups from the external subunits. MMM showed interaction distances of 1.55 and 1.63 Å; GGG of 1.63 and 1.71 Å; GMM of 1.52 and 1.58 Å; and MGM of 1.58 and 1.60 Å. GMG and GGM on the other hand, form only one electrostatic interaction with the carboxylate from the subunit in the middle, with an interaction distance of 1.54; and 1.63 Å; respectively. GGG, GGM, GMG, and MGM form one hydrogen bond. For the cases of GGG and GMG it is between the COO-Ethyl moiety from AML and a hydroxyl group from the G subunit. For GGM and MGM, the hydrogen bond is formed between one proton from the ammonium group of AML and a hydroxyl group from ALG.

### 3.8. Characterization of Oral Films Containing AML Nanoplex

Oral films were formulated as an innovative pharmaceutical dosage form using HPMC as the film-forming polymer, with incorporation of AML/ALG-MV nanoplexes. Table 5 summarizes the characteristics of the prepared films, which exhibited low thickness and a uniform weight distribution. The mean film thickness (47 µm) was within the range generally considered acceptable for orodispersible formulations (40–140 µm) [65]. The relative standard deviation of film weight was 7.1%, below the commonly recommended limit of 7.5% for this dosage form, and no individual unit deviated by more than 15% [66]. With respect to disintegration time, orodispersible films are generally required to disintegrate within 60 s to ensure adequate drug release. The prepared films exhibited a mean disintegration time of 34 s, thereby complying with this criterion [65].

The mean pH of the films was 7.1, which falls within the physiological range of saliva (pH 6.2–7.6) [67]. This is clinically relevant, as a near-neutral pH minimizes alterations in the oral environment upon film contact, thereby reducing the potential for mucosal irritation or discomfort [66]. With respect to mechanical performance, the films exhibited a mean tensile strength (TS) of 15.8 MPa and a mean elongation at break (EB) of 38.6%. When compared with the acceptance criteria commonly reported for the critical quality attributes of orodispersible film (TS of 15–35 MPa and an EB of 5–40%), the prepared films demonstrated suitable mechanical properties.

### 3.9. Dissolution of Amlodipine Polymeric Film

**Figure 9:**
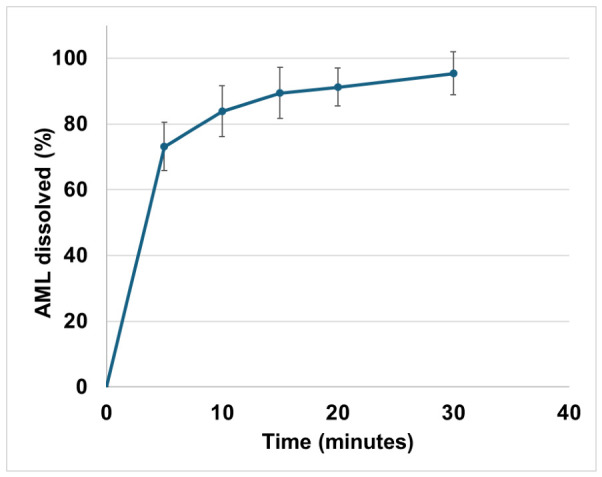
Dissolution profile of AML Oral Film.

The dissolution profile of the HPMC film incorporating AML/ALG-MV nanoplexes is presented in Figure 9. The film exhibited an immediate and rapid drug release profile, with approximately 73% of the drug dissolved within the first 5 min. Drug release proceeded at a high rate, reaching approximately 89% at 15 min, after which the dissolution curve began to plateau, approaching near-complete release (~95%) by 30 min. According to the USP monograph for amlodipine tablets [57], not less than 80% of the drug must be dissolved within 30 min; therefore, the oral film formulation satisfied the compendial dissolution requirement.

## 4. Discussion

The formation of AML–ALG nanoplexes was strongly dependent on both amlodipine concentration and alginate viscosity. At an AML concentration of 3 mg/mL, the dispersions exhibited pronounced turbidity along with the presence of visible solid aggregates, indicating not only nanoplex formation but also aggregation processes associated with reduced colloidal stability. In the study reported by S. Shahin et al. [36], precipitation was observed at an antimicrobial peptide–to–alginate mass ratio of 2:1, whereas optimal nanoplex formation was achieved at a 1:1 mass ratio. It is noteworthy that the cationic peptide, with an isoelectric point of 11.6 and a molecular weight of 2209.8 g/mol, differs substantially from amlodipine (408.9 g/mol), and that the physicochemical characteristics of the alginate used in that study were not reported. To address aggregation-related issues, a range of stabilizing agents has been reported in the literature. B. Dong et al. [68] enhanced the stability of ciprofloxacin–dextran nanoplexes through the incorporation of hydroxypropyl methylcellulose (HPMC, MW = 26 kDa) and polyvinylpyrrolidone (PVP, MW = 40 kDa), with HPMC demonstrating superior performance in prolonging physical stability and improving drug solubility. HPMC was also shown to be effective in nanoplexes formed between anionic curcumin and cationic chitosan, exhibiting superior performance compared with a co-amorphous curcumin–tannic acid complex [69]. In this study, the incorporation of Pluronic F127 at 0.1% (*w*/*v*) enabled the formation of stable nanoplexes at high AML:ALG mass ratios. Cheow et al. [70] employed Pluronic F68 at a concentration of 0.2% (*w*/*v*) for the same purpose during the formation of ciprofloxacin-dextran nanoplexes. Both substances (Pluronic F127 and F68) are members of the poloxamer family, a class of amphiphilic block copolymers that provide steric stabilization, thereby preventing close particle-particle interactions and subsequent aggregation [71,72]. An enthalpic stabilization mechanism has been proposed, whereby polyoxyethylene chains adsorbed onto the nanoparticle surfaces become hydrated in aqueous solution through hydrogen bonding with ethylene oxide groups. Interpenetration and compression of these ethylene oxide chains require the displacement of bound water molecules; however, this process requires an input of energy associated with a positive enthalpy change and is therefore thermodynamically non-spontaneous [73]. Consequently, this mechanism favors particle separation and contributes to colloidal stability.

Regarding the salt effect, the presence of Na^+^ and Cl^−^ ions attenuates the electrostatic interactions between the drug and the polymer. This screening effect leads to the formation of smaller nanoparticles, which consequently exhibit reduced light scattering. Kutscher et al. demonstrated that low salt concentrations (0.1 M) enhance nanoplex formation when ciprofloxacin is employed as a model drug [34]. A high salt concentration (1 M NaCl) leads to reduced encapsulation efficiency.

Salt concentration also influences nanoplex size, as NaCl at 0.1 and 0.3 M has been reported to induce a significant reduction in particle size compared with control conditions [34]. A similar trend was observed in the present study. In addition, nanoplex size was affected by both the molecular weight and block composition of alginate. Specifically, low-viscosity alginate (lower molecular weight) and alginates enriched in GGG blocks yielded the smallest nanoplexes. Yang et al. reported that Pickering emulsions prepared using sodium alginate with a lower M/G ratio (0.56) exhibited smaller particle sizes. Furthermore, calcium release studies conducted under simulated gastric conditions demonstrated a reduced calcium release ratio compared with alginates possessing a higher M/G ratio [74]. Another key parameter in particle size distribution analysis is the Polydispersity Index (PDI). In the context of drug delivery, a PDI value of 0.3 or below is generally regarded as acceptable, indicating a uniform and homogeneous population of nanoparticles [75,76]. The majority of values remained below this threshold; furthermore, the incorporation of Pluronic F127 in combination with 0.1 M NaCl significantly enhanced particle uniformity, yielding PDI values in the range of 0.195–0.271. In IL-6G (*Lessonia trabeculata*) nanoplexes, the elevated guluronic acid (G-block) content facilitates the formation of rigid, highly organized cooperative binding domains, thereby contributing to improved uniformity in particle assembly. Additional improvements in particle size distribution may be attained through the implementation of microfluidic production systems, which have been reported to exhibit superior performance compared to conventional nanoprecipitation techniques in terms of both particle size and PDI [77].

Computational simulations indicated that all evaluated models exhibited strongly interaction energies (>∆E_int_ −20 kcal/mol), confirming the formation of thermodynamically stable complexes between AML and the tested ALG sequences. These findings indicate that both M and G acid blocks are capable of effectively interacting with and binding AML. In addition, the ∆E_int_ reveals a preferential binding within the microenvironment represented by the GGG model, which is primarily attributable to the dominant contribution of electrostatic interactions (∆V_elstat_). It also showed the shortest electrostatic interaction distance. The following contribution comes from the polarization effects in the ALM-GGG complex, where GGG also presents the highest magnitude. The dispersion forces, although low in magnitude, reflect the high spatial complementary between AML and GGG, clearly denoted form the NCI analysis. This high complementarity would also be involved in the adoption of the shortest electrostatic interaction, working both contributions cooperatively. Considering that each polymer model is constituted by three subunits with a total of three carboxylate groups, the differences in the binding affinity relies on the spatial distribution of the rest of the system to accommodate AML into the polymer microenvironment, which consequently are determined by the dispersion interactions. The high magnitude of ∆E_Pauli_ for GGG results from the close proximity between AML and GGG, which is compensated by the stabilizing contributions to the interaction. These models were confirmed by infrared spectroscopy, as evidenced by the disappearance of absorption bands associated with the amino group of AML and the emergence of hydrogen-bonding interactions, indicated by a broad band corresponding to O-H stretching vibrations. A similar phenomenon has been reported for ciprofloxacin in dextran-based nanoplexes [32,69]. Additionally, in the nanoplexes, shifts in the absorption bands associated with the carboxyl groups of alginate were observed, consistent with ionic interactions, as previously reported in the literature [42,78].

With respect to innovative pharmaceutical dosage forms of AML, a dry emulsion system exhibiting a controlled release profile for up to 120 min has been reported [18]. In the same study, the free drug achieved only 50% release at 60 min, reflecting the poor solubility of amlodipine base. A comparable result was observed in the present study. Furthermore, the proposed innovative pharmaceutical dosage form, formulated as an oral film, exhibited a rapid release profile that complies with the requirements of the United States Pharmacopeia. Although formal in vitro–in vivo correlation (IVIVC) studies have not been established, bioequivalence assessments demonstrate that generic amlodipine tablet formulations achieve plasma concentration–time profiles comparable to those of the reference (innovator) product, while also exhibiting similar in vitro dissolution characteristics under United States Pharmacopeia conditions [79].

## 5. Conclusions

Alginate viscosity and M/G ratio are critical parameters for modulating nanoplex size and the release kinetics of amlodipine. The HPMC film incorporating nanoplexes demonstrated high drug entrapment efficiency combined with rapid dissolution, indicating a promising strategy to improve amlodipine delivery and potentially enhance its bioavailability. Characterization results show that the ALG-MV nanoplex-loaded HPMC film exhibits robust mechanical properties, including adequate strength and flexibility, together with rapid functional performance, supporting its suitability for patient administration. Nevertheless, further studies are required to assess oral permeation and to determine the formulation’s capacity to achieve a rapid therapeutic effect.

## Figures and Tables

**Table 1:** Characterization of Amlodipine.

Test	Result
^1^H NMR	Consistent with structure (δ): 7.35 (dd,1H), 7.26 (dd, 1H), 7.23 (td, 1H), 7.14–7.10 (m, 1H), 5.29 (s, 1H), 4.63 (d, 1H), 4.53 (d, 1H), 4.02–3.91 (m, 2H), 3.50 (s, 3H), 3.44 (t, 2H), 2.73 (t, 2H), 2.30 (s, 3H), 1.10 (t, 3H).
ATR-IR	Consistent with structure (cm^−1^): 3389, 1682, 1646, 1601, 1473, 1308, 1276, 1202 and 1035.
MS-ESI+	Consistent with structure: ESI+ ion peak = 409.17 *m*/*z* Theoretical ion calculated = 409.14 *m*/*z* (based on monoisotopic mass: 408.14 Da)
DSC	Crystalline solid with endothermic event (Onset = 138.4 °C/Peak = 140.6 °C)
Solubility	0.11 ± 0.01 mg/mL in Water at 37 °C

**Table 2:** Characterization of the Alginate samples.

Sample Code	Seaweed	M/G Ratio	ATR-IR Bands (cm^−1^)	Viscosity (mPa·s)
ALG-LV	*Macrocystis pyrifera*	1.41	1596, 1404, 944, 881	11.5 ± 0.3 (22 °C)
ALG-MV	*Macrocystis pyrifera*	1.59	1596, 1404, 944, 881	1306.2 ± 5.1 (22 °C)
IL-6F	*Lessonia berteroana*	1.28	1596, 1405, 944, 884	140.9 ± 0.5 (22 °C)
IL-6G	*Lessonia trabeculata*	0.62	1599, 1407, 947, 902, 809, 779	183.7 ± 0.8 (22 °C)

**Table 3:** Encapsulation Efficiency, particle size and Zeta potential of nanoplexes.

Sample Code	EE (%)	Particle Size (nm)	PDI	Zeta Potential (ζ) (mV)
AML/ALG-LV 1/1 ratio	80.1 ± 1.2	302.2 ± 7.3	0.317 ± 0.02	−28.8 ± 1.2
AML/ALG-LV 1/2 ratio	84.5 ± 0.5	313.4 ± 15.9	0.379 ± 0.04	−34.7 ± 0.6
AML/ALG-MV 1/1 ratio	82.3 ± 0.8	404.3 ± 19.7	0.438 ± 0.02	−32.0 ± 0.4
AML/ALG-MV 1/2 ratio	86.7 ± 0.3	442.1 ± 14.9	0.207 ± 0.03	−38.3 ± 0.4
AML/ALG-LV 3/1 ratio NaCl 0.1 M-PF127	82.3 ± 1.4	316.3 ± 3.4	0.195 ± 0.02	−24.4 ± 0.2
AML/ALG-LV 3/2 ratio NaCl 0.1 M-PF127	87.4 ± 0.5	453.1 ± 15.4	0.240 ± 0.02	−32.3 ± 0.7
AML/ALG-MV 3/1 ratio NaCl 0.1 M-PF127	83.8 ± 1.1	465.8 ± 17.2	0.258 ± 0.05	−29.1 ± 2.1
AML/ALG-MV 3/2 ratio NaCl 0.1 M-PF127	89.5 ± 0.3	539.8 ± 25.4	0.271 ± 0.04	−33.6 ± 0.8
AML/ALG-LV 3/2 ratio NaCl 0.3 M-PF127	85.1 ± 1.0	339.9 ± 21.2	0.292 ± 0.07	−29.8 ± 0.6
AML/ALG-MV 3/2 ratio NaCl 0.3 M-PF127	87.5 ± 0.5	446.3 ± 16.9	0.318 ± 0.04	−31.0 ± 0.5
AML/IL-6F 1/2 ratio	85.3 ± 0.7	394.8 ± 26.4	0.268 ± 0.04	−34.4 ± 2.0
AML/IL-6G 1/2 ratio	89.2 ± 0.9	322.0 ± 28.4	0.205 ± 0.05	−33.2 ± 0.5

**Table 4:** Comparative evaluation of dissolution profiles based on the similarity factor (f_2_).

Sample Code	Similarity Factor (f_2_)				
	ALG-LV	ALG-MV	IL-6F	IL-6G	AML
AML	18	20	22	25	NA
IL-6G	43	48	62	NA	25
IL-6F	46	58	NA	62	22
ALG-MV	50	NA	58	48	20
ALG-LV	NA	50	46	43	18

NA: not applicable.

**Table 5:** Characterization of HPMC film incorporating AML/ALG-MV nanoplexes.

Test	Result
Film thickness	47 ± 2 µm
Weight uniformity	19.4 ± 1.4 mg (RSD = 7.2)
Disintegration time	34 ± 6 s (s)
pH	7.1 ± 0.1
Tensile strength (TS)	15.8 ± 0.9 MPa
Elongation at break (EB)	38.6 ± 2.7%

RSD: relative standard deviation.

## Data Availability

The data supporting the findings of this study are provided in the paper and supporting information.

## Supplementary Materials

The following supporting information can be downloaded at https://www.mdpi.com/article/10.3390/pharmaceutics18060653/s1: Figure S1: ^1^H-NMR spectroscopy experiment of Amlodipine; Figure S2: FT-IR spectroscopy experiment of Amlodipine; Figure S3: (a) Mass spectrum of Amlodipine. (b) Thermoanalytical curve of Amlodipine; Figure S4: The anomeric region in the 400 MHz—1H NMR spectrum of Sodium Alginate from *Macrocystis pyrifera* low-viscosity (ALG-LV), Sodium Alginate from *Macrocystis pyrifera* medium-viscosity (ALG-MV), Sodium alginate *Lessonia berteroana* (IL-6F) and Sodium alginate from *Lessonia trabeculata* (IL-6G); Figure S5: FT-IR spectroscopy experiment of Sodium Alginate from *Macrocystis pyrifera* low-viscosity, Sodium Alginate from *Macrocystis pyrifera* medium-viscosity, Sodium alginate *Lessonia berteroana* and Sodium alginate from *Lessonia trabeculata*. Figure S6: Graphical representation of the electrostatic and hydrogen bonds formed between the AML-ALG complexes.

## Abbreviations

The following abbreviations are used in this manuscript:

AML	amlodine free-base form
ALG	alginate
M	mannuronic acid
G	guluronic acid
M/G	mannuronic acid to guluronic acid ratio
ALG-LV	low-viscosity alginate derived from *Macrocystis pyrifera*
ALG-MV	medium-viscosity alginate derived from *Macrocystis pyrifera*
IL-6F	alginate derived from *Lessonia berteroana*
IL-6G	alginate derived from *Lessonia trabeculata*
