# Supplementary material for: Development of a Novel Alginate-Based Amlodipine Nanoplex for the Formulation of an Oral Film in Antihypertensive Therapy
Source: Pharmaceutics. 2026 May 27;18(6):653. doi: 10.3390/pharmaceutics18060653 (PMC13304620; doi:10.3390/pharmaceutics18060653)
Supplement: Supplementary file 1 [file pharmaceutics-18-00653-s001.zip › pharmaceutics-4254731-supplementary.pdf]

pharmaceutics

Supplementary Material

Development of a Novel Alginate-Based Amlodipine Nanoplex for the Formulation of an Oral Film in Antihypertensive Therapy

Javiera Medina [1], Thamara Hidalgo [1], Fabián Martínez [1], María Elena Gamboa-Arancibia [1], Néstor Gutiérrez-Sánchez [2], Sebastián Miranda-Rojas [2] and Alexander Gamboa [1,*]

1 Departamento de Ciencias del Ambiente, Facultad de Química y Biología, Universidad de Santiago de Chile, Av. Libertador Bernardo O'Higgins 3363, Estación Central, Santiago 9170022, Chile;
2 Departamentode Ciencias Químicas, Facultad de Ciencias Exactas, Universidad Andrés Bello, Av. República 275, Santiago 8370146, Chile
* Correspondence: alexander.gamboa@usach.cl; Tel.: +56-2-2-7181159

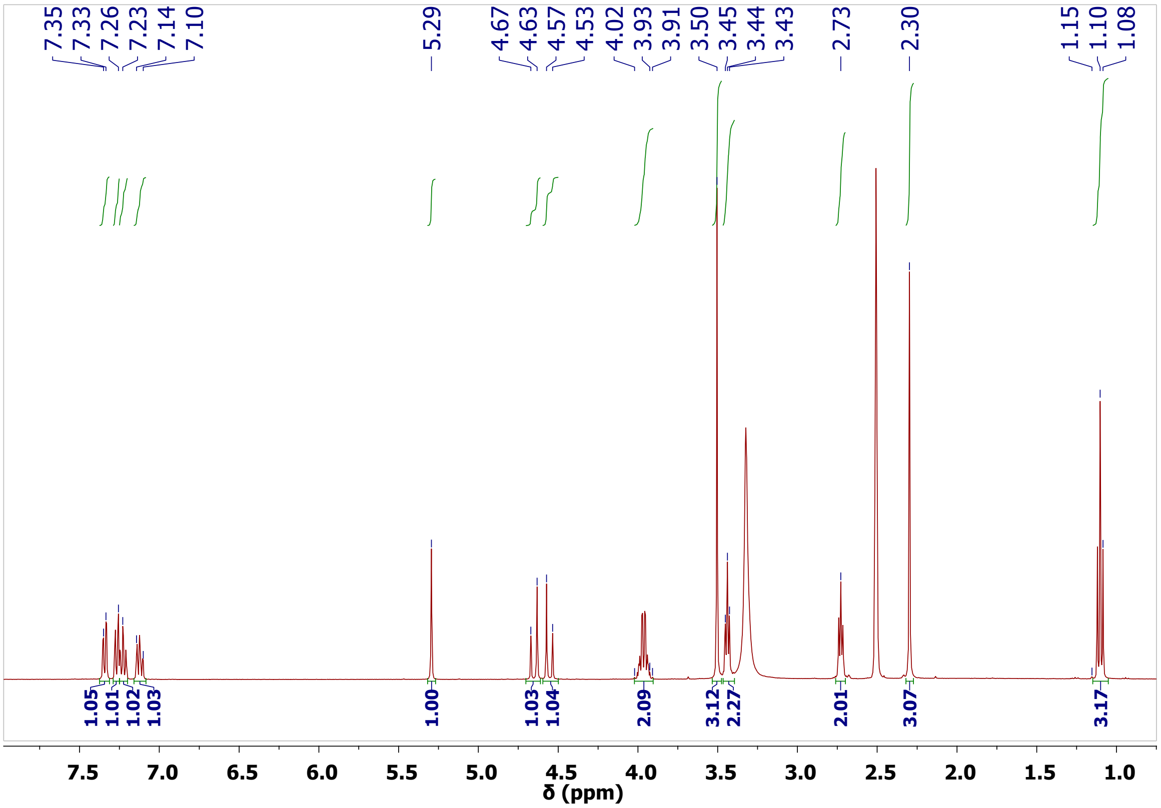

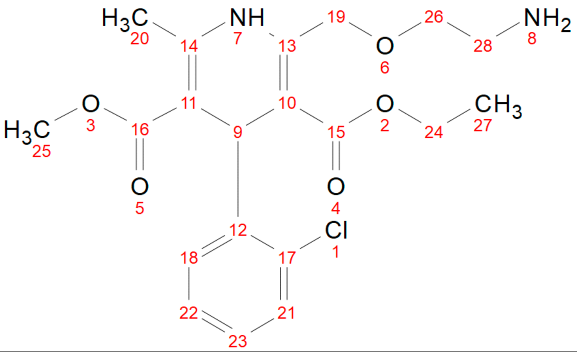

Proton	δ (ppm)	Proton	δ (ppm)
H18	7.35 (dd,1H)	H24	4.02-3.91 (m, 2H)
H21	7.26 (dd, 1H)	H25	3.50 (s, 3H)
H22	7.23 (td, 1H)	H26	3.44 (t, 2H)
H23	7.14-7.10 (m, 1H)	H28	2.73 (t, 2H)
H9	5.29 (s, 1H)	H20	2.30 (s, 3H)
H19	4.63 (d, 1H) 4.53 (d, 1H)	H27	1.10 (t, 3H)

Figure S1. ^{1}H-NMR spectroscopy experiment of Amlodipine.

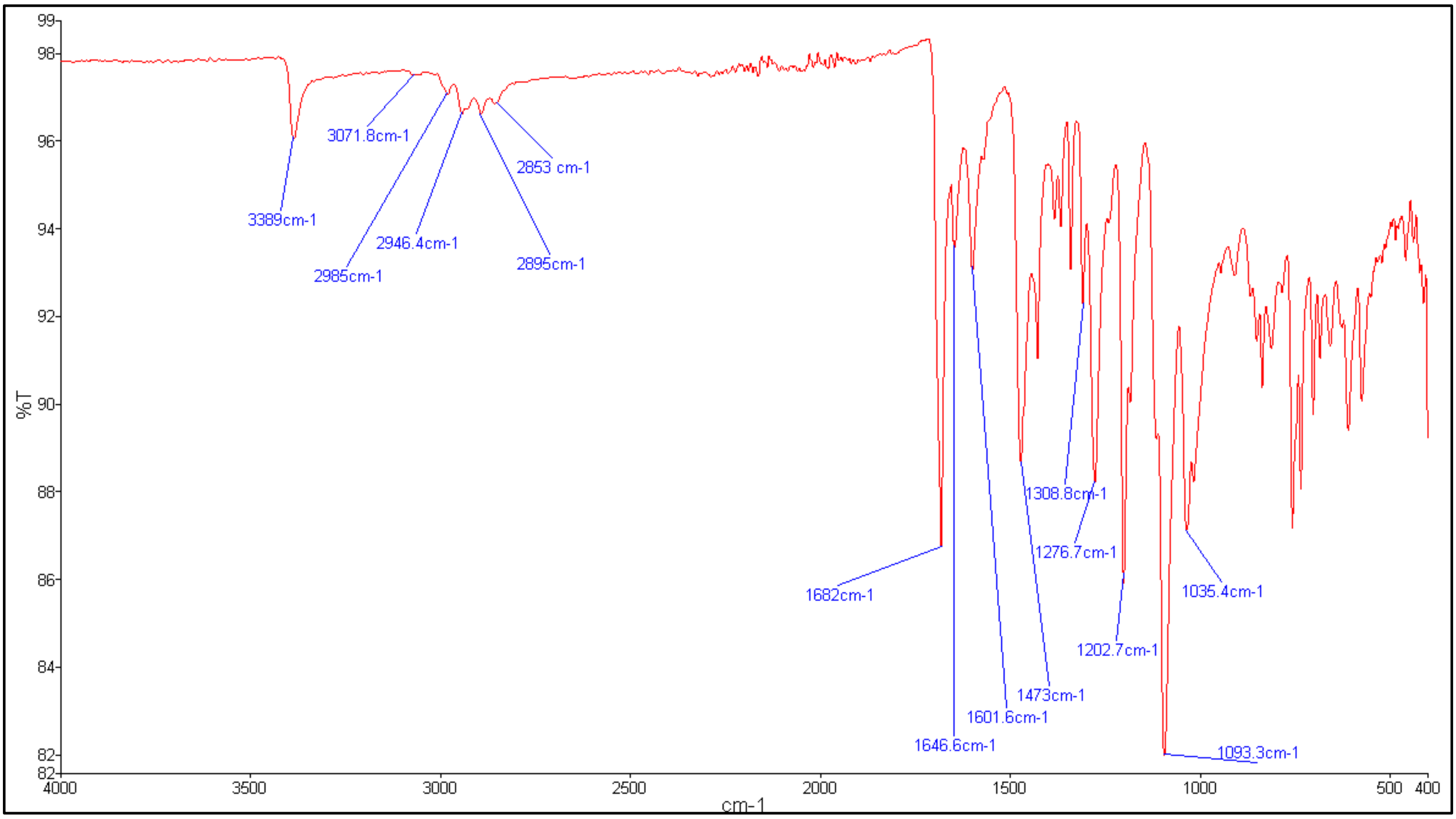

Figure S2. FT-IR spectroscopy experiment of Amlodipine.

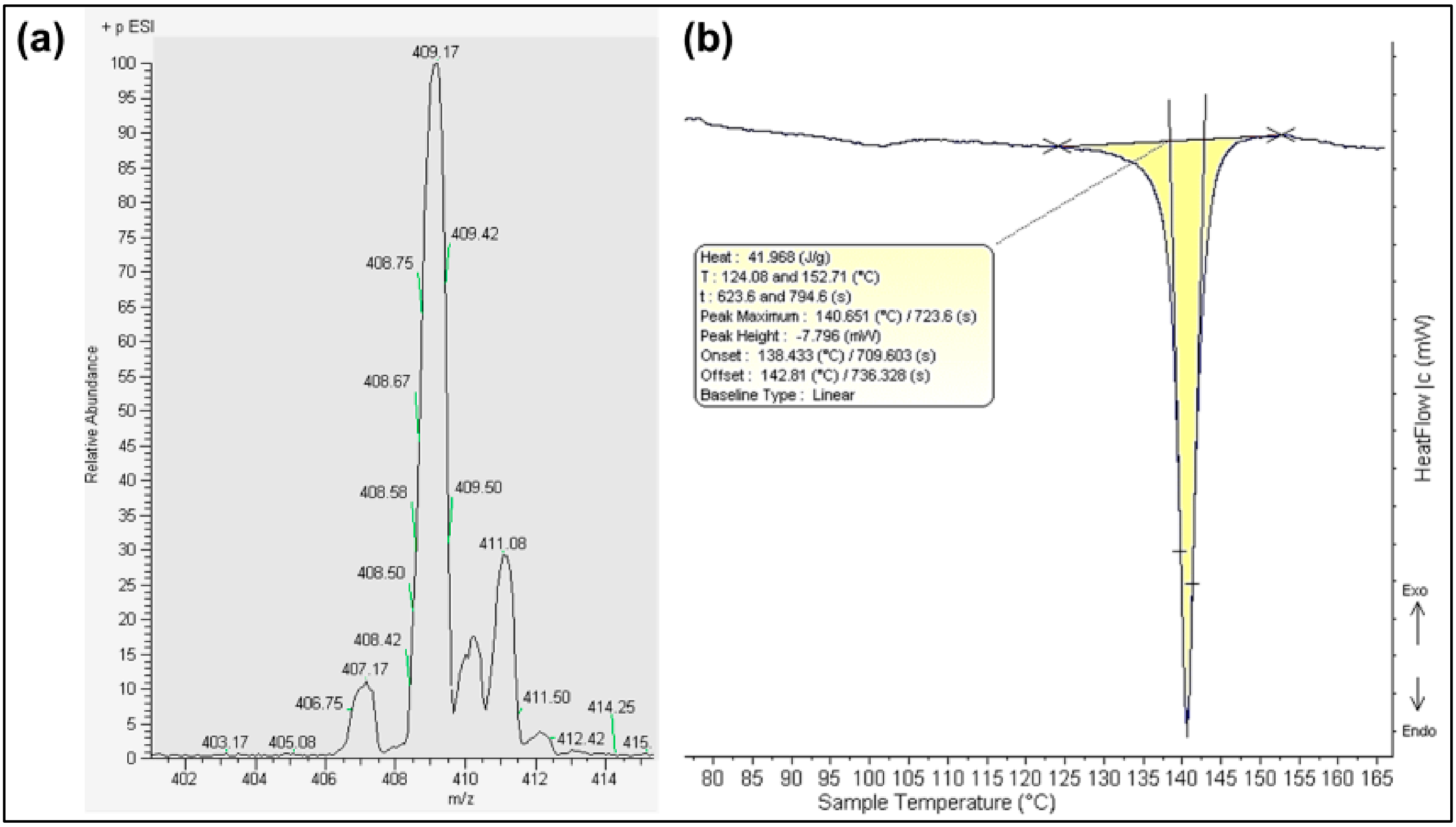

Figure S3. (a) Mass spectrum of Amlodipine. (b) Thermoanalytical curve of Amlodipine

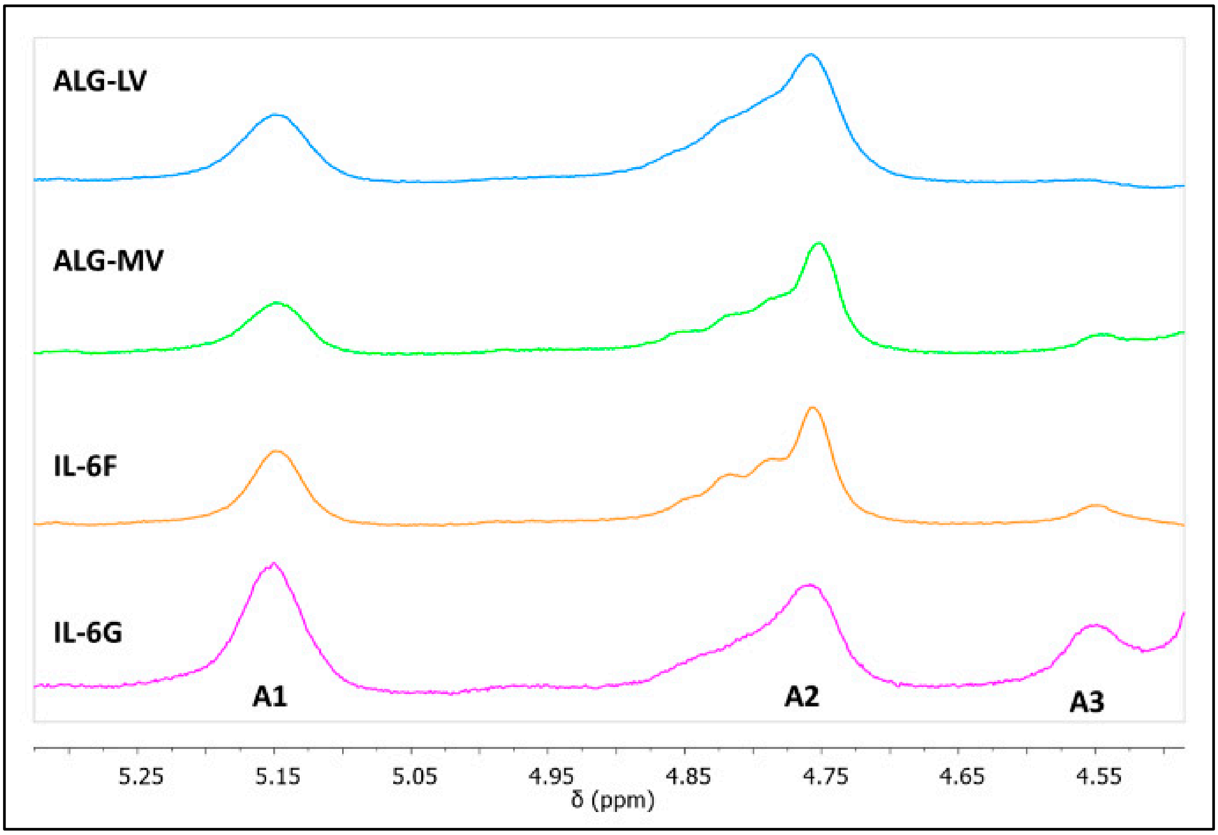

Figure S4. The anomeric region in the 400 MHz - ^{1}H NMR spectrum of Sodium Alginate from *Macrocystis pyrifera* low-viscosity (ALG-LV), Sodium Alginate from *Macrocystis pyrifera* medium-viscosity (ALG-MV), Sodium alginate *Lessonia berteroana* (IL-6F) and Sodium alginate from *Lessonia trabeculata* (IL-6G). **FG =A1/(A2 + A3); FM = 1 – FG; M/G = (1 – FG) / FG.**

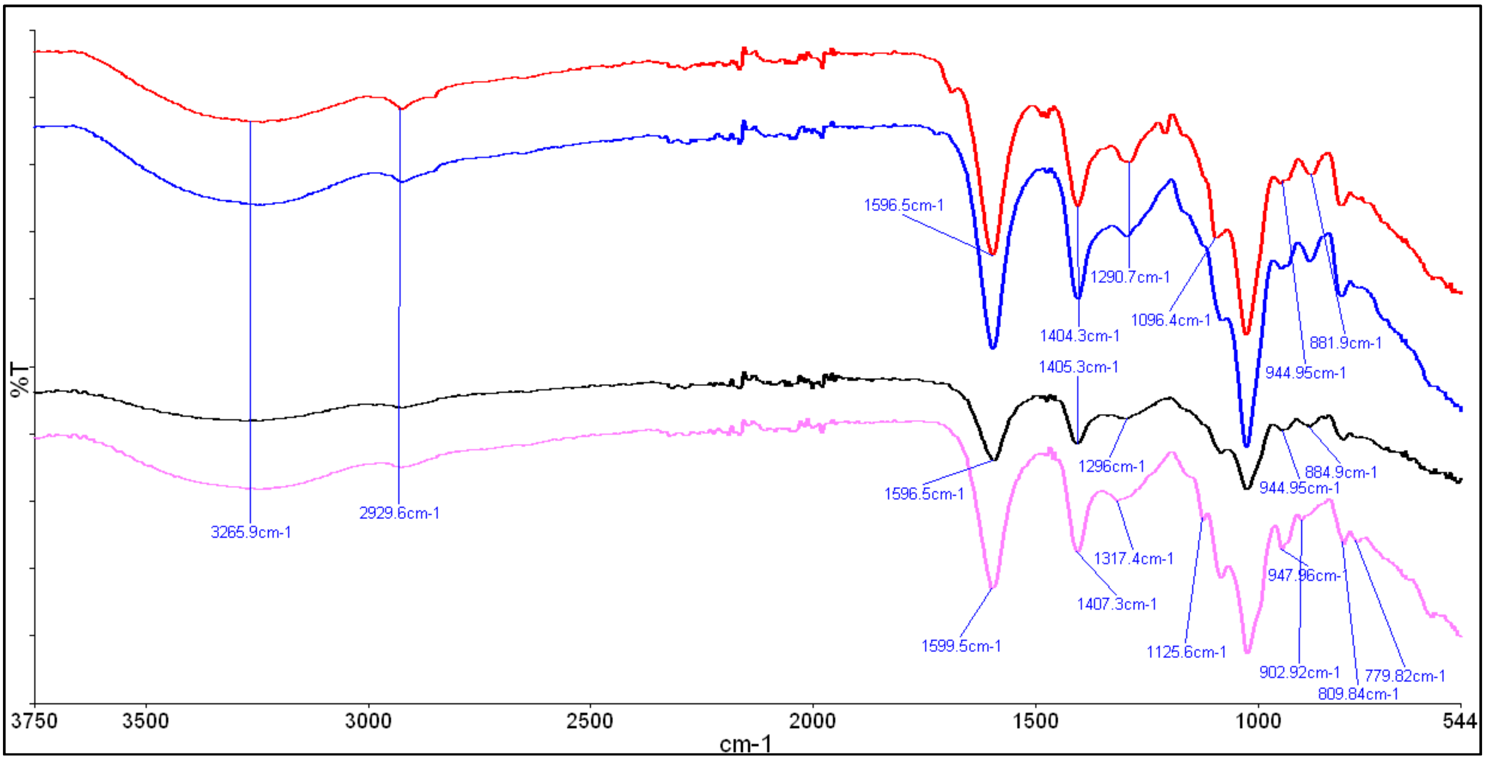

Figure S5. FT-IR spectroscopy experiment of Sodium Alginate from *Macrocystis pyrifera* low-viscosity (red), Sodium Alginate from *Macrocystis pyrifera* medium-viscosity (blue), Sodium alginate *Lessonia berteroana* (black) and Sodium alginate from *Lessonia trabeculata* (pink).

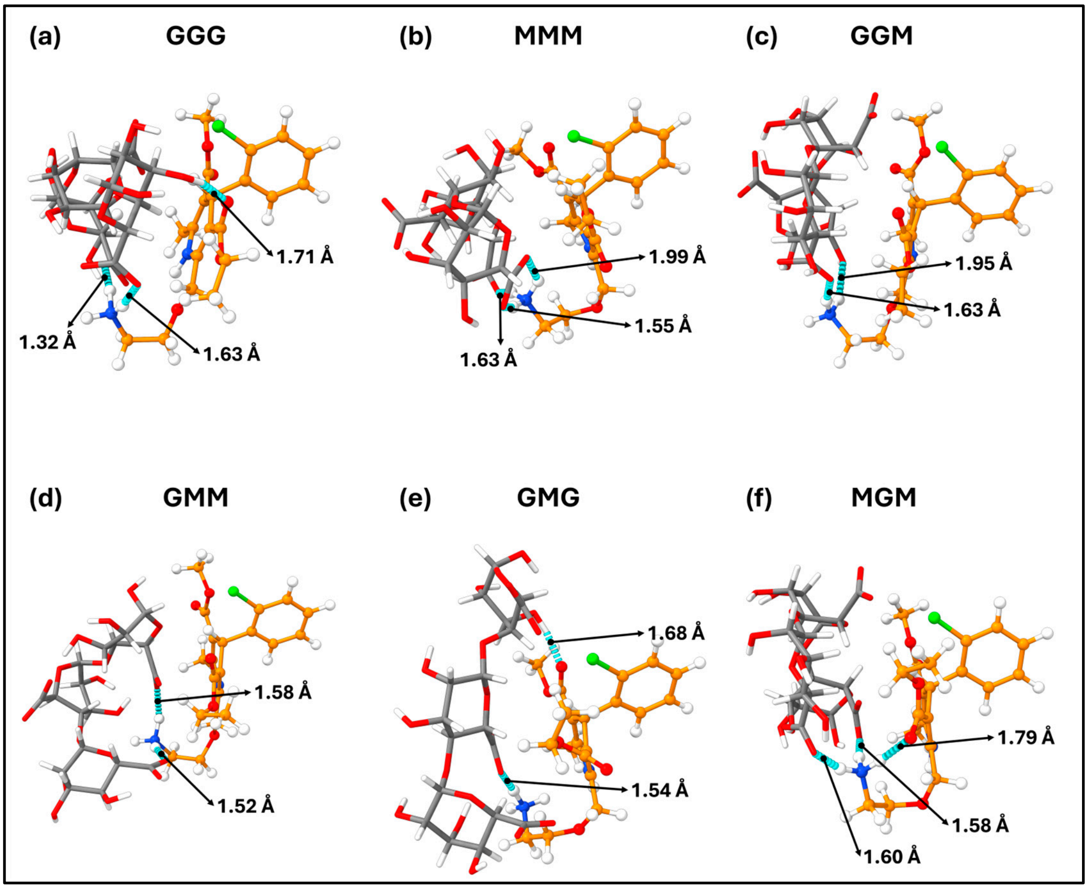

Figure S6. Graphical representation of the electrostatic and hydrogen bonds formed between the AML-ALG complexes.
